# Potential Role of Acacia Senegal (Gum Arabic) as Immunomodulatory Agent among newly diagnosed COVID 19 Patients: A structured summary of a protocol for a randomised, controlled, clinical trial

**DOI:** 10.1186/s13063-020-04707-2

**Published:** 2020-09-05

**Authors:** Lamis Kaddam, Rasha Babiker, Sara Ali, Shahinaz Satti, Nour Ali, Maha Elamin, Mowaia Mukhtar, Mustafa Elnimeiri, Amal Saeed

**Affiliations:** 1 Department of Physiology, Faculty of Medicine, Alneelain University Khartoum, P.O. Box: 11121, 12702 Khartoum, Sudan; 2 Present Address: Department of Physiology, Faculty of Medicine, King Abdul-Aziz University, Rabigh, Saudi Arabia; 3 Department of Physiology, Faculty of Medicine, National University-Sudan, Khartoum, Sudan; 4 Department of Microbiology, Faculty of Medical Laboratory Sciences, International University of Africa, Khartoum, Sudan; 5 Department of Physiology, Faculty of Medicine, Nile University, Khartoum, Sudan; 6 Department of Biochemistry, Faculty of Medicine, Alneelain University, Khartoum, Sudan; 7 Institute of Endemic Diseases, Faculty of Medicine, University of Khartoum, Khartoum, Sudan; 8 Department of Community Medicine, Faculty of Medicine, Alneelain University, Khartoum, Sudan; 9 Department of Physiology, Faculty of Medicine, University of Khartoum, Khartoum, Sudan

**Keywords:** COVID-19, Randomised controlled trial, protocol, Gum Arabic, cytokines, Immunomodulation

## Abstract

**Objectives:**

To investigate the potential efficacy of Acacia Senegal extract Gum Arabic (GA) supplementation as immunomodulatory and anti-inflammatory dietary intervention among newly diagnosed COVID 19 Sudanese patients. To study the effect of GA on the level of cytokines, TNFα, IL8, IL6 IL10, CRP and the viral load.

Secondary outcomes will be the effect of GA oral intake on mortality rate and days of hospital admission.

**Trial design:**

Quadruple blind, randomized placebo-controlled clinical trial Phase II & III.

Prospective, two-arm, parallel-group, randomised (1:1 allocation ratio) superiority trial of oral GA among seropositive COVID-19 patients.

**Participants:**

Inclusion criteria:

COVID-19 infected (newly diagnosed) as proved by real-time PCR within 72 hours of PCR.

Age 8-90 years

Both genders

Exclusion criteria:

Intubated patients on parenteral treatment

Allergy to Gum Arabic

The study will be conducted in COVID Isolation Centres and Soba University Hospital Khartoum State Sudan.

**Intervention and comparator:**

Experimental: Intervention Group

This arm will receive 100% natural Gum Arabic provided in a powder form in 30-grams-dose once daily for four weeks

Placebo Comparator: Control group: This group will be provided with pectin powder provided as one-gram-dose once daily for four weeks

Both GA and placebo will be in addition to standard care treatment based on local clinical guidelines.

**Main outcomes:**

Mean change from baseline score of Immune Response to end of the trial. Changes of the level of Tumor Necrosis Factor (TNFα), interleukin IL8, IL6, and IL10 from the baseline values (Four weeks from the start of randomization).

Mortality rate: The percentage of deaths among COVID 19 patients received Gum Arabic compared to placebo (Four weeks from the start of randomization]).

**Randomisation:**

Randomization (1:1 allocation ratio) and will be conducted using a sequence of computer-generated random numbers by an independent individual.

Each participating centre will be assigned a special code generated by the computer. The randomization will be kept by the PI and a research assistant.

**Blinding (masking):**

Quadruple (Participant, Care Provider, Investigator, Outcomes Assessor)

**Numbers to be randomised (sample size):**

110 eligible patients will be randomly assigned to either GA (n=55) or placebo (n=55) groups.

**Trial Status:**

Protocol Version no 2, 30^th^ June 2020. Recruitment will start on 15^th^ September 2020. The intended completion date is 15^th^ January 2021.

**Trial registration:**

ClinicalTrials.gov Identifier: NCT04381871. Date of trial registration: 11 May 2020.

**Full protocol:**

The full protocol is attached as an additional file, accessible from the Trials website (Additional file 1). In the interest in expediting dissemination of this material, the familiar formatting has been eliminated; this Letter serves as a summary of the key elements of the full protocol.

## Supplementary information

**Additional file 1.** Full Study Protocol.

## Data Availability

The datasets used and/or analysed during the current study will be available from the corresponding author on reasonable request

