# Supplementary material for: Potential Role of Acacia Senegal (Gum Arabic) as Immunomodulatory Agent among newly diagnosed COVID 19 Patients: A structured summary of a protocol for a randomised, controlled, clinical trial
Source: Trials. 2020 Sep 5;21:766. doi: 10.1186/s13063-020-04707-2 (PMC7474312; doi:10.1186/s13063-020-04707-2)
Supplement: Supplementary file 1 — Additional file 1. Full Study Protocol. [file 13063_2020_4707_MOESM1_ESM.pdf]

	National Medicines & Poisons Board
**Version No.: 2**<br>**Version Date: 30.6,2020**<br>**Revision No.: *-1***<br>**Revision Date: 13.5.2020**<br>**Code No: TQM/PIr-F/4**	***Clinical Trials and Drug Research on Human and Animal Application Form***

For office use only

Proposal No.:

Date Received:

00

Part 1:

1. **Principal investigator (PI) / Applicant Attachment Relevant C.V**

Name: Dr Lamis Kaddam
• Institute: Alneelain university
• Qualification<br>Phd in physiology
Experience in research<br>Experience Human Physiology<br>Gum Arabic clinical trials<br>Researches in Hematology
• Current position:<br>Assistant professor of Physiology
• Address / e-mail:

<u>e-mail : lamiskaddam@hotmail.com</u><br><br>Office Tel. Mobile Tel.0912979736
• Post Office Tel.<br>Mobile Tel.0912979736 | Mobile Tel.
Signature :

2. Co-investigator (1)

• Name: Dr. Rasha Babiker
• Institute:<br>• Physiology Department, Faculty of Medicine National University
• Qualification PhD in physiology
• Experience in research<br>Experience in Human Physiology<br><br>Gum Arabic clinical trials
• Current position:<br>Assistant professor
• Address / e-mail:<br>e-mail :rashat33@yahoo.com
• Post Office Tel. Tel. | Mobile Tel. 0912951517
Signature :

2. Co-investigator (2)

Name:<br><br>**Dr. Sara Mohammed Ali**
• Institute: Microbiology Department, Faculty of Medical laboratory Sciences International University of Africa

• Qualification PhD in microbiology
• Experience in research<br>• Molecular biology<br>• Microbiology
• Current position:<br>• University of Africa<br>• Department of Microbiology
Address / e-mail: e-mail: saraali@iua.edu.sd

• Post Office Tel. Office Tel.<br>Mobile Tel. 0901910142<br>•	Mobile Tel.
Signature :	

2. Co-investigator (3)

Name: Dr .Shahenaz Seifaldeen Mustafa Satti<br>• Mobile Tel. 0912204825
• Institute:<br>Alneelain University Faculty of Medicine Physiology Department
• Qualification<br>Msc human physiology - PhD student
• Experience in research<br>Experience in research Physiology<br>Medical Education<br>Gum Arabic<br>Community based researches
• Current position:<br>Lecturer o Physiology
• Address / e-mail:shahenazsatti@gmail,com

post Office Tel. Mobile Tel. 0912204825	Mobile Tel.
• Signature:	

Co- investigator 4

**Name: Professor Amal Mahmoud Saeed**
• Institute:<br>Khartoum University - Faculty of Medicine- Physiology Department
• Qualification<br>Professor . phd physiology
Experience in Human Physiology<br><br>Medical Education<br><br>Gum Arabic clinical trials
• Current position:<br>Professor in University of Khartoum Faulty Of Medicine
Address / e-mail: amalsaeed@yahoo.com

post Office Tel. Mobile Tel.	Mobile Tel.
• Signature:	

Co investigator 5

**Name: Professor Mustafa Khidir Elnimeiri**
• Institute:<br>Alneelain university Faculty of Medicine-
• Qualification

• MD Community medicine Professor of Preventive Medicine & Epidemiology- -Khartoum-Sudan-National Consultant of TB & HIV/AIDS and Reproductive Health.
• Experience in research<br>• Preventive Medicine<br>• Community based surveys<br>• Research methodology, research ethics, scientific writing and medical education.
• Current position:<br>Assistant Dean for Post-graduate and Scientific Research-Faculty of Medicine-Alneelain University
• Address / e-mail:<br>• E-mail: nimeirimust@gmail.com
Mobile : 0912353275
• Signature:

co investigator 6

**Dr.Nour Elkhair Ali Khalaf allah**<br>Physiology Department, Faculty of Medicine ,Nile University
Title of post, position or appointment presently held by co- investigator(4)<br>Complete Postal address / e-mail: Nooralkhair@gmail.com<br>Office Tel. Mobile Tel. 0911288100

Co-Investigator 7

Name: Professor Mowaia Mukhtar
• Institute: Institute of Endemic diseases University of Khartoum
• Qualification<br>Phd in Immunology and Molecular Biology
Experience in research<br><br>Immunology<br>Molecular biology<br>Microbiology
• Current position:<br>Professor
• Address / e-mail:<br>e-mail : mmukhatr@tropmedicine.org<br><br>Office Tel. Mobile Tel. 50912315828
Signature :

Co-Investigator 7

Name:Dr. Maha Ismail Elamin
• Institute: Alnelain University faculty of Medicine Department of Biochemistry
• Qualification<br>MBBS, MSC PhD in Medical Biochemistry,
Experience in research<br><br>Clinical Nutrition<br>Molecular biology<br>Medical Education
• Current position:<br>Associate Professor, Biochemistry and Molecular Biology, Assistant Dean for Quality and Development Faculty of Medicine Alneelain University.
• Address / e-mail:<br>e-mail : drmaha59@gmail.com<br><br>Office Tel. Mobile Tel. 0912230895
Signature :

Part2

1. Title of Proposal:

Protective effect of Gum Arabic (*Acacia Senegal*) supplementation on immune responses and inflammatory markers among COVID 19 Patients

Double Randomized two arms clinical trial

Introduction:

COVID 19 Coronavirus disease (COVID-19) is caused by SARS-COV2 and represents the causative agent of a potentially fatal disease caused global public concern. The disease spreads rapidly compared to the previous pandemics, which makes it more difficult to contain. Due to this, there is an urgent need for fast diagnosis, preventive and therapeutic measures. Patients infected with COVID-19 showed higher leukocyte numbers, abnormal respiratory findings, and increased levels of plasma pro-inflammatory cytokines. Main mechanism for ARDS (Acute Respiratory Distress Syndrome) is the cytokine storm, uncontrolled systemic inflammatory response resulting from the release of pro-inflammatory cytokines. The cytokine storm will trigger a violent attack by the immune system to the body, cause ARDS and multiple organ failure, and finally death in severe cases. Current investigations, demonstrated that T cell numbers are negatively correlated to serum IL-6, IL-10 and TNF-α concentration, and proposed T cells as potential therapeutic trget.

Butyrate, is an epigenetic regulator via its capacity to act as a histone deacetylase (HDAC) inhibitor. This may be directly relevant to the modulation of covid-19 infection. Although data on the biological interactions of covid-19 with human physiology have still to be published, HDAC inhibition is a common regulator of the effects of many viruses, including the influenza virus. The inhibition o fHDAC1, as induced by butyrate, decreases influenza-driven pneumonia infections.

Gum Arabic (GA) is a safe dietary fibres approved by FDA and WHO. It is high molecular weight polysaccharide retrieved from Acacia Senegal and Acacia Seyal trees. GA fermentation produced short chain fatty acids mainly propionate and butyrate. Gum Arabic has anti-inflammatory effect

which has been investigated in various diseases and conditions.

Scientific Background:

COVID 19 Pathogenesis:

COVID 19 Coronavirus disease (COVID-19) is caused by SARS-COV2 and represents the causative agent of a potentially fatal disease that is of great global public health concern(1, 2).

Patients infected with COVID-19 showed higher leukocyte numbers, abnormal respiratory findings, and increased levels of plasma pro-inflammatory cytokines(1-3). Additionally, a value of 16.16 mg/L of blood C-reactive protein was noted which is above the normal range (0–10 mg/L)(2). Patients infected with COVID-19 showed higher leukocyte numbers, abnormal respiratory findings, and increased levels of plasma pro-inflammatory cytokines. Significantly high blood levels of cytokines and chemokines were noted in patients with COVID-19 infection that included IL1-β, IL1RA, IL7, IL8, IL9, IL10, basic FGF2, GCSF, GMCSF, IFNγ, IP10, MCP1, MIP1α, MIP1β, PDGFB, TNFα, and VEGFA(1) The latest report shows the number of CD4+ and CD8+ T cells in the peripheral blood of SARS-CoV-2-infected patients significantly is reduced, Similarly, the acute phase response in patients with SARS-CoV is associated with severe decrease of CD4+ T and CD8+ T cells(3). The report in Lancet shows ARDS is the main death cause of COVID-19. Of the 41 SARS-CoV-2-infected patients admitted in the early stages of the outbreak, six died from ARDS(3). Main mechanism for ARDS is the cytokine storm, the deadly uncontrolled systemic inflammatory response resulting from the release of large amounts of pro-inflammatory cytokines (3).The cytokine storm will trigger a violent attack by the immune system to the body, cause ARDS and multiple organ failure, and finally lead to death in severe cases of SARS-CoV-2 infection(1, 2) . IFN-I(IFN-α and IFN-β) has a protective effect on SARS-CoV and MERS-CoV infection, but the IFN-I pathway is inhibited in infected mice(4). n Number of total T cells, CD4+ and CD8+ T cells were dramatically reduced in COVID-19 patients, especially among elderly patients (≥60 years of age) and in patients requiring Intensive Care Unit (ICU)(3). Statistical analysis demonstrated that T cell numbers are negatively correlated to serum IL-6, IL-10 and TNF-α concentration, potential therapeutic targets preventing cytokine storm.

Gum Arabic mechanism of action:

Gum Arabic fermentation increased the level of serum butyrate. Butyrate, including in its nutraceutical form, sodium butyrate, is an epigenetic regulator via its capacity to act as a histone deacetylase (HDAC) inhibitor. This may be directly relevant to the modulation of covid-19 infection(5). Although data on the biological interactions of covid-19 with human physiology have still to be published, HDAC inhibition is a common regulator of the effects of many viruses, including the influenza virus. The inhibition of HDAC1, as induced by butyrate, decreases influenza-driven pneumonia infections(5)

Gum Arabic (GA) is a safe dietary fibres approved by FDA and WHO(6). It is high molecular

weight non-starch polysaccharide retrieved from Achaia Senegal and Achaia Seyal trees(6, 7). GA is mainly fermented by colonic bacteria instead of been digested among humans(8) and animals (6, 7). GA fermentation produced short chain fatty acids mainly propionate and butyrate (6-8). The latter discovered as physiological modifier for different body function like modulation of cell proliferation, apoptosis, regulation of angiogenesis and inflammation(9). Gum Arabic anti-inflammatory effect has been investigated in various diseases and conditions (10-12). GA exerted local anti-inflammatory effects by modifying nuclear factor-κB (NF-κB) on the small intestine(13).

Gum Arabic as anti-inflammatory agent:

Gum Arabic anti-inflammatory effect has been investigated in clinical trials (10,12). An anti-inflammatory effect of GA was demonstrated via the application of GA in drinking water given to rats with diabetes mellitus by a decrease of TNFα and IL-1β and increase of IL-10 (14). Interestingly, Bovo et al. investigated the effect of GA on the complement system, as arabinogalactans are reported to be complement system modulators. The authors showed a pro-inflammatory activity of GA by activation of the classical and alternative pathways of the complement system (15). GA exerted local anti-inflammatory effects by modifying nuclear factor-κB (NF-κB) on the small intestine (12). In addition, a reduction of oxidative stress was shown after GA supplementation of drinking water in the liver tissue of rats with type I diabetes (16) and oral GA supplementation in humans suffering from sickle cell anemia (17). GA significantly increased oxidative burst/ROS production of bovine and human granulocytes in a dose-dependent manner (18,19). Similarly, arabinogalactan–protein (AGP) of GA may have pro-inflammatory properties on certain parts of the immune system(18, 20).

From all the above the main pathogenesis of COVID 19 is due to inflammatory process and cytokine storm. Gum Arabic is a prebiotic dietary fibers with proven anti-inflammatory properties and immunemodulatory agent. Nowadays, most of research have carried out on corona-virus are in the primary stages and the exact mechanisms remains unknown, infection spread and deaths are increasing in daily bases, the urge for vaccine and protective elements against the immunological response of the body against the virus is needed.

Justification:

A novel corona virus from Wuhan in central China, named 2019- nCoV, has recently caused an epidemic of pneumonia in humans and posed a huge threat to global public health. To the Date according to national ECDPC -nCoV has led to more than 2744744 cases including 195387 deaths. Which put heath system word wide in great challenge because no licensed vaccine and specific treatment. Identification of natural products with direct antimicrobial effects or also immunomodulatory effects to boost the innate immune defense is a promising strategy to identify and characterize new treatment or prevention strategies. GA is a well-known traditional herbal medication from Acacia Senegal. Gum Arabic is prebiotic agent, with immune modulator properties. Enhancing natural immunity and ameliorate the cytokines storm, will be of great

benefits for COVID 19 patients to overcome the disease.

3. Objectives

General objective:

Explore the role of Gum Arabic as immune modulator among COVID 19 Sudanese patients.

Specific objectives:

1- To understand the immune response of disease progression and physiological pathway in patients consuming Gum Arabic (Acacia Senegal) for one month.

2- To study the innate and acquired mechanisms of defense against the coronavirus (cytokine and antibody responses) in placebo and experimental groups in each.

3- To asses clinical progression of the disease in group treated with Gum Arabic (Acacia Senegal)

4- Measure the level of anti-inflammatory marker (CRP & TNFα, IL6, IL10, IFγ) before and after GA ingestion

4. Methodology

- Study Plan: (Phases, Activities)

Phase III clinical trial

Method:

Study design: Double blind, randomized placebo controlled clinical trial Phase III.

The study will be conducted on patients with COVID 19 infection for 4 weeks.

Study setting: The study will be conducted in Khartoum Isolation centers and Soba hospital Khartoum State Sudan

Sample size: All Patients who presented to Gabra and Soba hospital and confirmed with COVID 19 during the study period will be included after satisfying the inclusion criteria

Study population: (inclusion and exclusion criteria)

(Inclusion criteria:)

1-COVID 19 infected as proved by real time PCR. (Newly diagnosed)

2-Age 8-90 years

3-Both gender

Exclusion criteria:

1-Intubated patients on parental treatment

2- Pregnancy

3- Gum Arabic allergy

Randomization and blinding:

The subjects, after stratification by age and sex, will randomly assigned to receive coded and indistinguishable Gum Arabic or placebo. Randomization will be conducted by using a sequence of computer-generated random numbers by independent individual.

Each participating center will be assigned a special code generated by the computer. The

randomization will be kept by the PI and a research assistant. The assessor for clinical outcomes would be blinded to the randomization status. The research staffs who perform the intervention would not know the assessment results. After the eligibility assessment by viral screening, laboratory and clinical assessments would be conducted at the baseline (after completion of informed consent), at first, second and 4th week

Gum Arabic administration:

GA in powder form, it is a 100% natural extract powder produced mechanically from the wildly grown Acacia Senegal tree with a particle size less than 210 µm. The daily dose is 45 gram per day. It will be given in one sachet to be consumed in the early morning dissolved in 200 ml of water. The GA will be provided to the participants weekly for four weeks (7 sachets for each visit). Standardized questionnaire and check list will be used to collect data about patients' physical examination, weight, height, severity of the symptoms and any side effects. Gum Arabic will be purchased from nature gum company, Khartoum Sudan.

Placebo will be pectin one gram per day. It will be given in one sachet to be consumed in the early morning dissolved in 200 ml of water for 4 weeks.

Investigations:

The procedures for screening will be performed within one week before dosing, except where otherwise stated, and included the following:

1- Medical history,

2- Physical examination, including weight, height and vital signs.

3- The following routine laboratory tests: (A) CBC (B) serum chemistry

including renal function tests (RFT) and Liver function test (LFT)

(serological tests)

i. **Real time PCR:** To determine the viral load
ii. **Flowcytometry:** To measure the CD4 counts.
iii. **ELISA for cytokines**

Study protocols:

Each patient will receive daily dose

The patients will be followed every week and the following investigation will be done

during the visit for follow up: CBC, and urine analysis. LFT and RFT will be done weekly

Blood samples will be collected by certified nurse and will be used solely for the research

purposes and will be destructed after completing the project.

Primary outcome:

Mean change from baseline score of Immune Response to end of the trial (Time Frame: up to 4 weeks)

Secondary outcome:

1-clinical improvement and hospital discharge [Time Frame: from the date of assignment until the date of hospital discharge for the last assigned case, whichever comes first, assessed up to two months]

2-Time of discharge from hospital after full recovery

5. Data analysis (Methods used for Data analysis)

Data will be collected and coded and entered in excel sheet.

SPSS .24 and Graph prism will be used. Paired *t* test will be used to determine statistical significance between baseline and post treatment results. *P* equal to or less than .05 will consider statistically significant. Data will be presented as means (±SD).

- **References:**

References:

1. Li X, Geng M, Peng Y, Meng L, Lu S. Molecular immune pathogenesis and diagnosis of COVID-19. Journal of Pharmaceutical Analysis. 2020.

2. Rothan HA, Byrareddy SN. The epidemiology and pathogenesis of coronavirus disease (COVID-19) outbreak. Journal of autoimmunity. 2020:102433.

3. Diao B, Wang C, Tan Y, Chen X, Liu Y, Ning L, et al. Reduction and functional exhaustion of T cells in patients with coronavirus disease 2019 (COVID-19). Medrxiv. 2020.

4. Deng X, Yu X, Pei J. Regulation of interferon production as a potential strategy for COVID-19 treatment. arXiv preprint arXiv:200300751. 2020.

5. Anderson G. Psychological Stress and Covid-19: Interactions with Gut Microbiome and Circadian Rhythm in Driving Symptom Severity.

6. Phillips GO, Ogasawara T, Ushida K. The regulatory and scientific approach to defining gum arabic (Acacia senegal and Acacia seyal) as a dietary fibre. Food hydrocolloids. 2008;22(1):24-35.

7. Patel S, Goyal A. Applications of natural polymer gum arabic: a review. International Journal of Food Properties. 2015;18(5):986-98.

8. Cherbut C, Michel C, Raison V, Kravtchenko T, Severine M. Acacia gum is a bifidogenic dietary fibre with high digestive tolerance in healthy humans. Microbial Ecology in Health and Disease. 2003;15(1):43-50.

9. Smith JG, Yokoyama WH, German JB. Butyric acid from the diet: actions at the level of gene expression. Critical Reviews in Food Science. 1998;38(4):259-97.

10. Kaddam LA, Kaddam AS. Effect of Gum Arabic (Acacia senegal) on C-reactive protein level among sickle cell anemia patients. BMC Research Notes. 2020;13(1):1-5.

11. Elamin S, Alkhawaja MJ, Bukhamsin AY, Idris MA, Abdelrahman MM, Abutaleb NK, et al. Gum arabic reduces C-reactive protein in chronic kidney disease patients without affecting urea or indoxyl sulfate levels. International journal of nephrology. 2017;2017.

12. Kamal E, Kaddam LA, Dahawi M, Osman M, Salih MA, Alagib A, et al. Gum Arabic Fibers Decreased Inflammatory Markers and Disease Severity

Score among Rheumatoid Arthritis Patients, Phase II Trial. International journal of rheumatology. 2018;2018.

13. Wapnir RA, Sherry B, Codipilly CN, Goodwin LO, Vancurova I. Modulation of rat intestinal nuclear factor NF-κB by gum Arabic. Digestive diseases and sciences. 2008;53(1):80-7.

14. Al Za'abi M, Al Salam S, Al Suleimani Y, Manoj P, Nemmar A, Ali BH. Gum Acacia improves renal function and ameliorates systemic inflammation, oxidative and nitrosative stress in streptozotocin-induced diabetes in rats with adenine-induced chronic kidney disease. Cell Physiol Biochem. (2018) 45:2293–304. doi: 10.1159/000488176

15. Bovo F, Lenzi RM, Yamassaki FT, Messias-Reason IJ, Campestrini LH, Stevan FR, et al. Modulating effects of arabinogalactans from plant gum exudates on human complement system. Scand J Immunol. (2016) 83:314–20. doi: 10.1111/sji.12427

16. Amanullah M. Protective effect of long term administration of gum Arabic on oxidative stress in hepatic tissue of diabetic rats. Biomed J Sci Tech Res. (2018) 4:1–7. doi: 10.26717/BJSTR.2018.04.0001110

17. Kaddam L, Fadl-Elmula I, Eisawi OA, Abdelrazig HA, Salih MA, Lang F, et al. Gum Arabic as novel anti-oxidant agent in sickle cell anemia, phase II trial. BMC Hematol. (2017) 17:4. doi: 10.1186/s12878-017-0075-y

18. Gamal el-din AM, Mostafa AM, Al-Shabanah OA., Al-Bekairi AM, Nagi MN. Protective effect of arabic gum against acetaminopheninduced hepatotoxicity in mice. Pharmacol Res. (2003) 48:631–5. doi: 10.1016/S1043-6618(03)00226-3

19. Al Za'abi M, Al Salam S, Al Suleimani Y, Manoj P, Nemmar A, Ali BH. Gum Acacia improves renal function and ameliorates systemic inflammation, oxidative and nitrosative stress in streptozotocin-induced diabetes in rats with adenine-induced chronic kidney disease. Cell Physiol Biochem. (2018) 45:2293–304. doi10.1159/000488176

20.Baien SH, Seele J, Henneck T, Freibrodt C, Szura G, Moubasher H, Nau R, Brogden G, Mörgelin M, Singh M, Kietzmann M, von Köckritz-Blickwede M and de Buhr N (2020) Antimicrobial and Immunomodulatory Effect of Gum Arabic on Human and Bovine Granulocytes Against Staphylococcus aureus and Escherichia coli. Front. Immunol. 10:3119. doi: 10.3389/fimmu.2019.03119

- **Annexes:**

-Ethics consideration

-Relevant C.V for Principal investigator

-Declaration of the authority responsible for research

-Declaration of Agreement for Participation in the Research

بروتوكول البحث -

الأخلاقية الإعتبارات -

الذاتية للباحث الاساسي السيرة

اقرار الجهة المسؤلة عن البحث -

إقرار موافقة للمشاركة في البحث-

استمارة تقييم الوحدات البحثية للجهات التي يسمح فيها إجراء -
التجارب الطبية

- إحضار عقد الجهة البحثية والجهة الممولة للبحث
